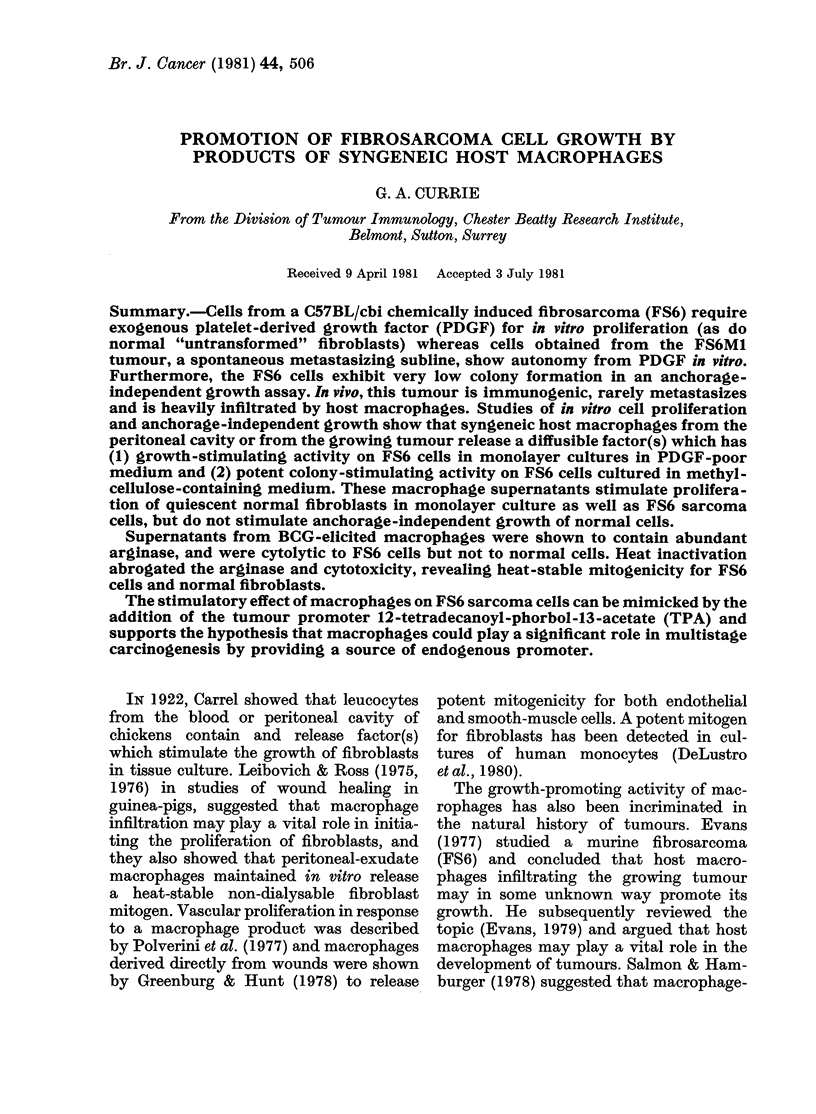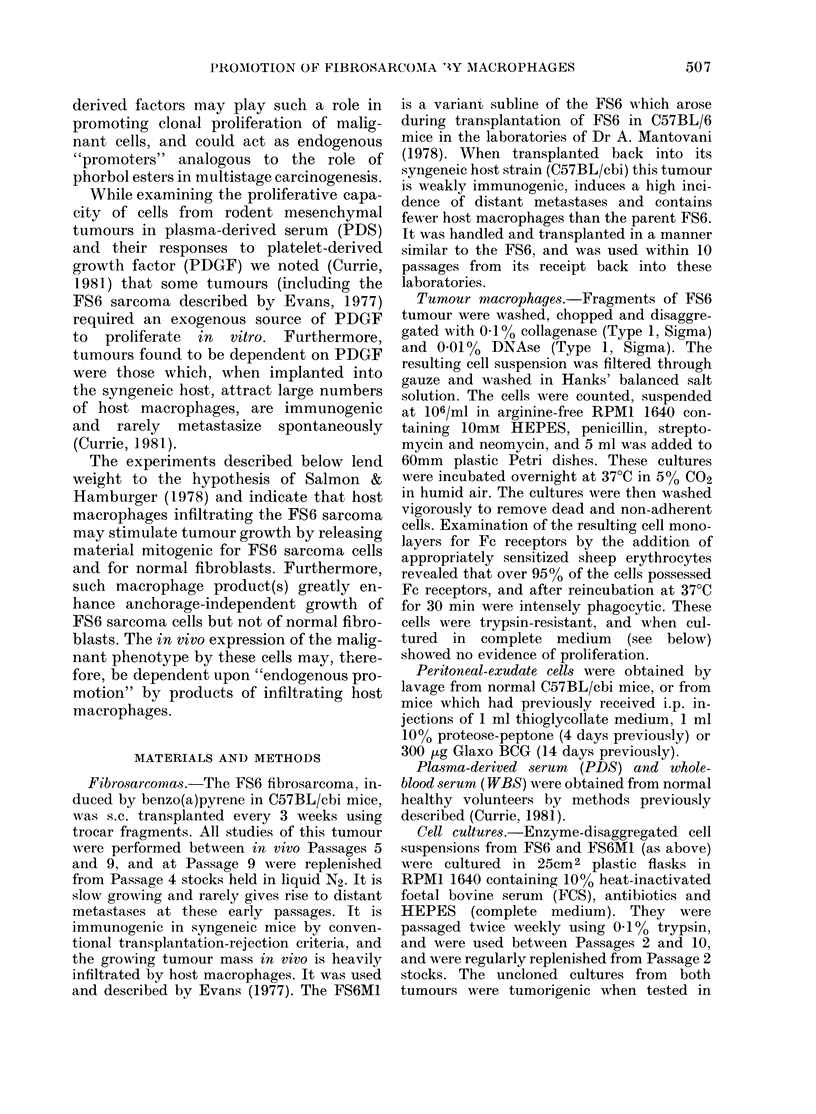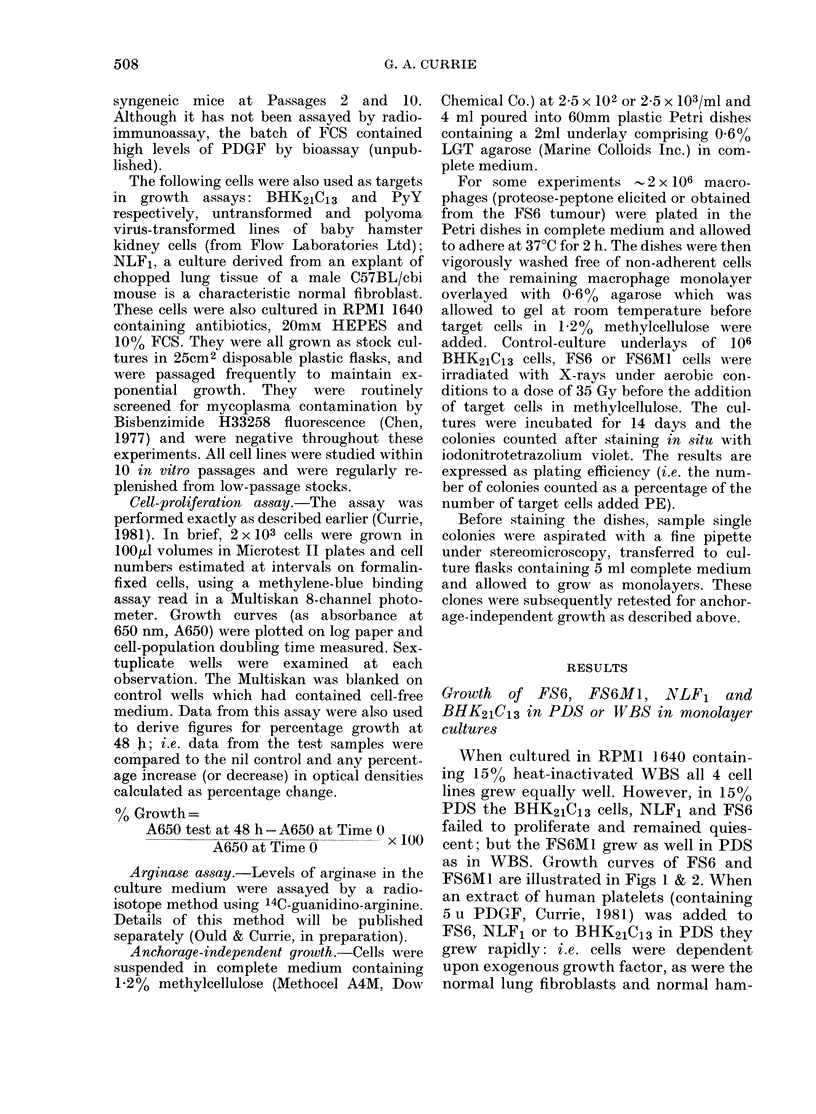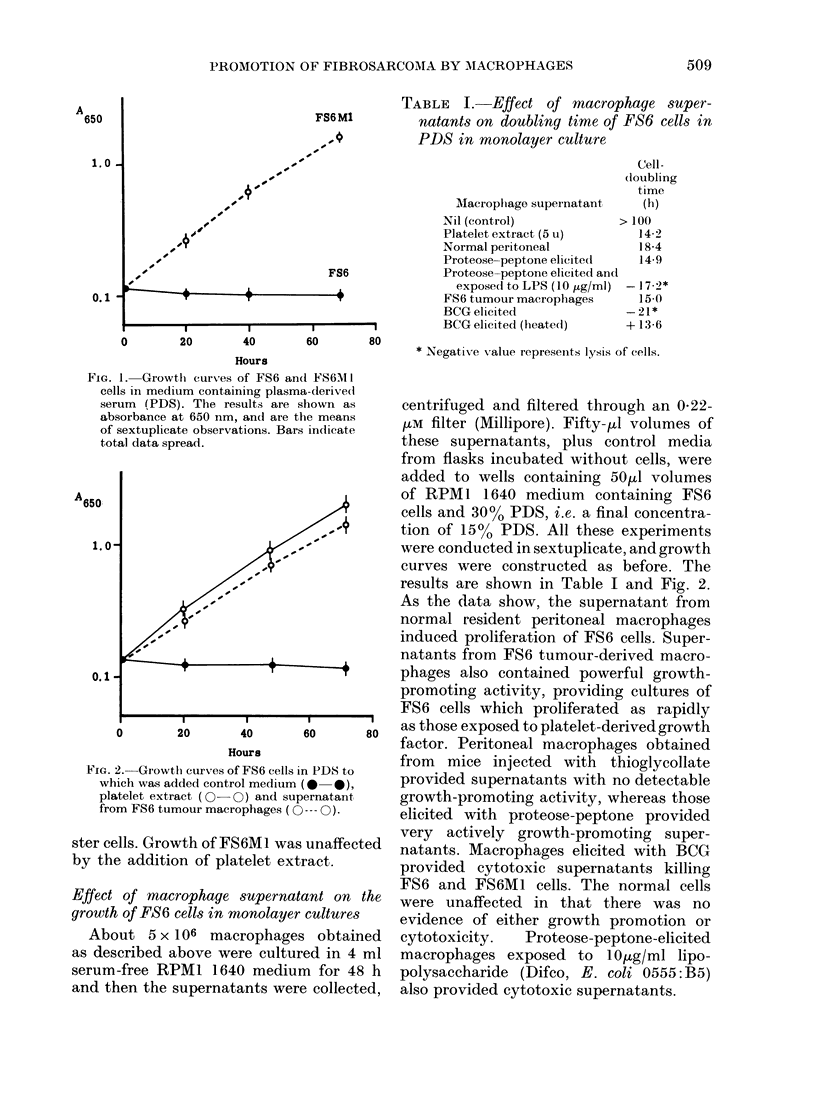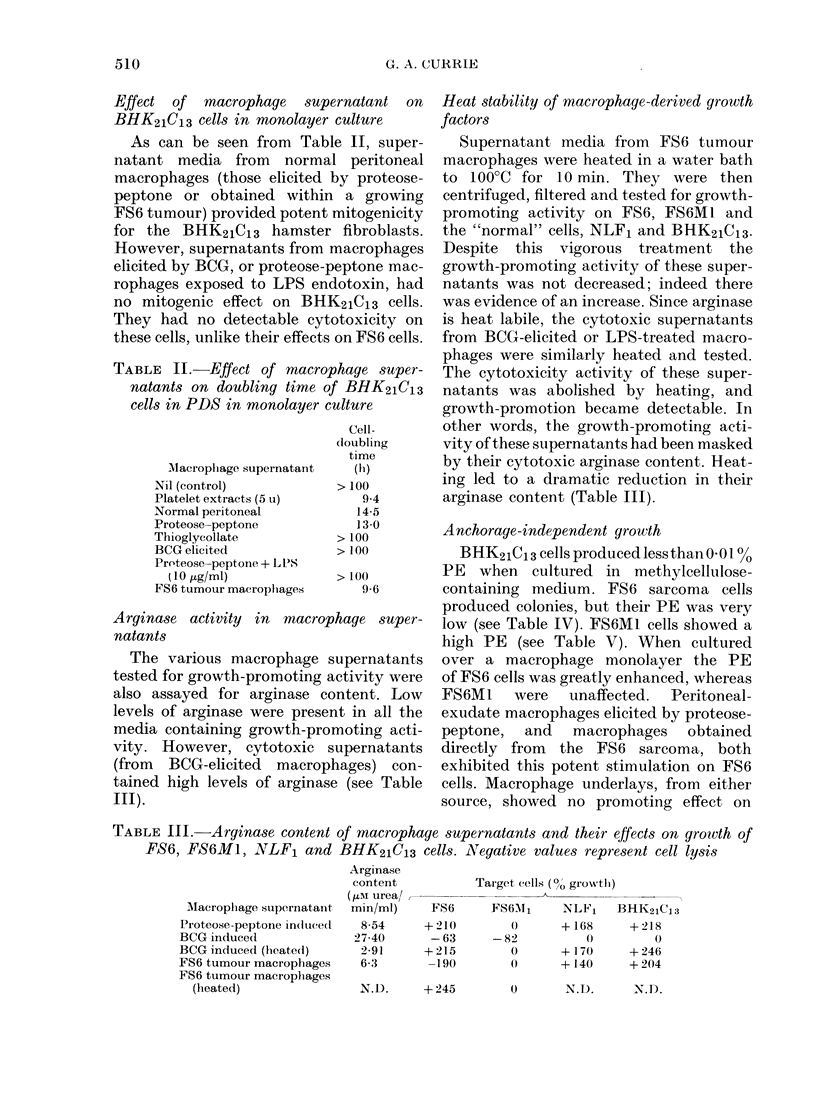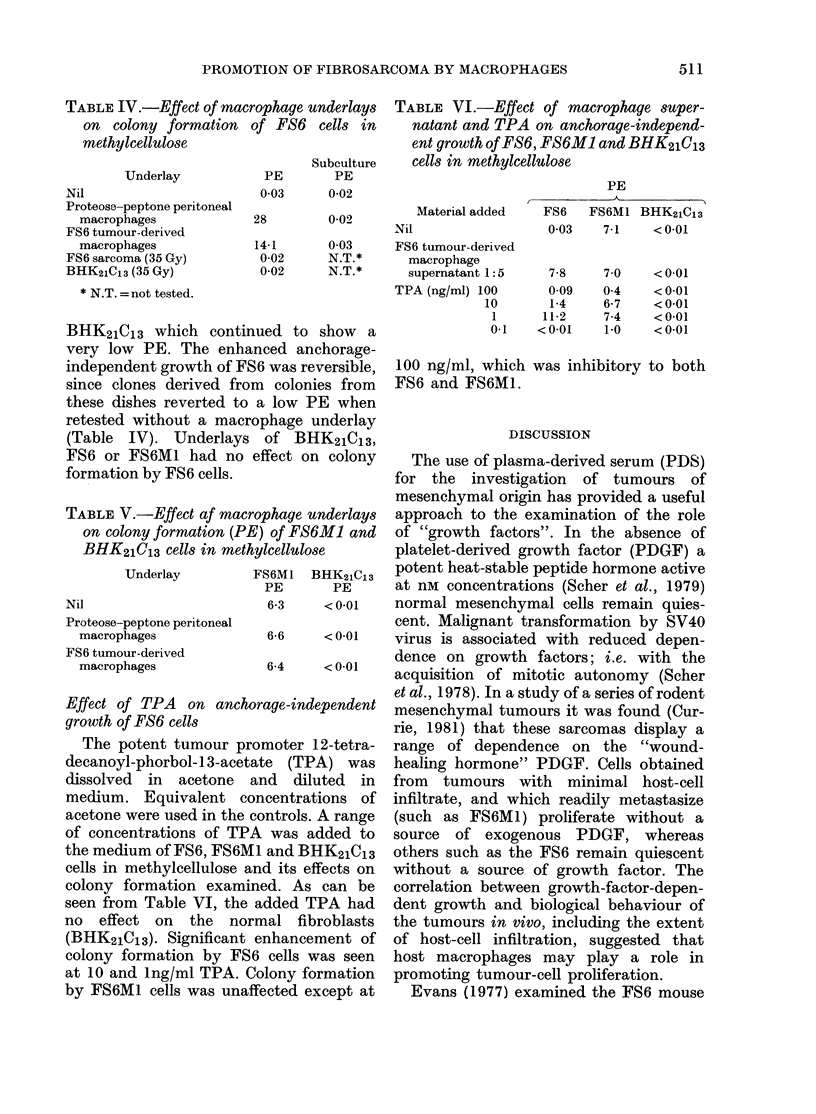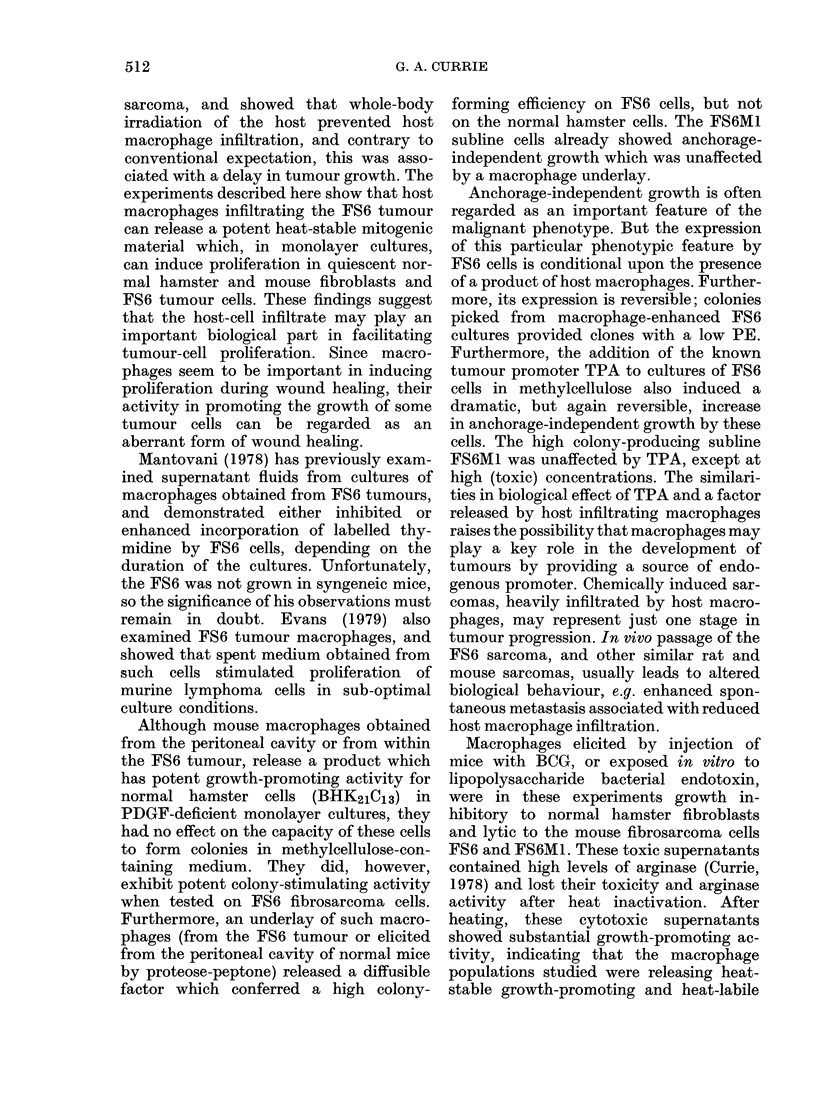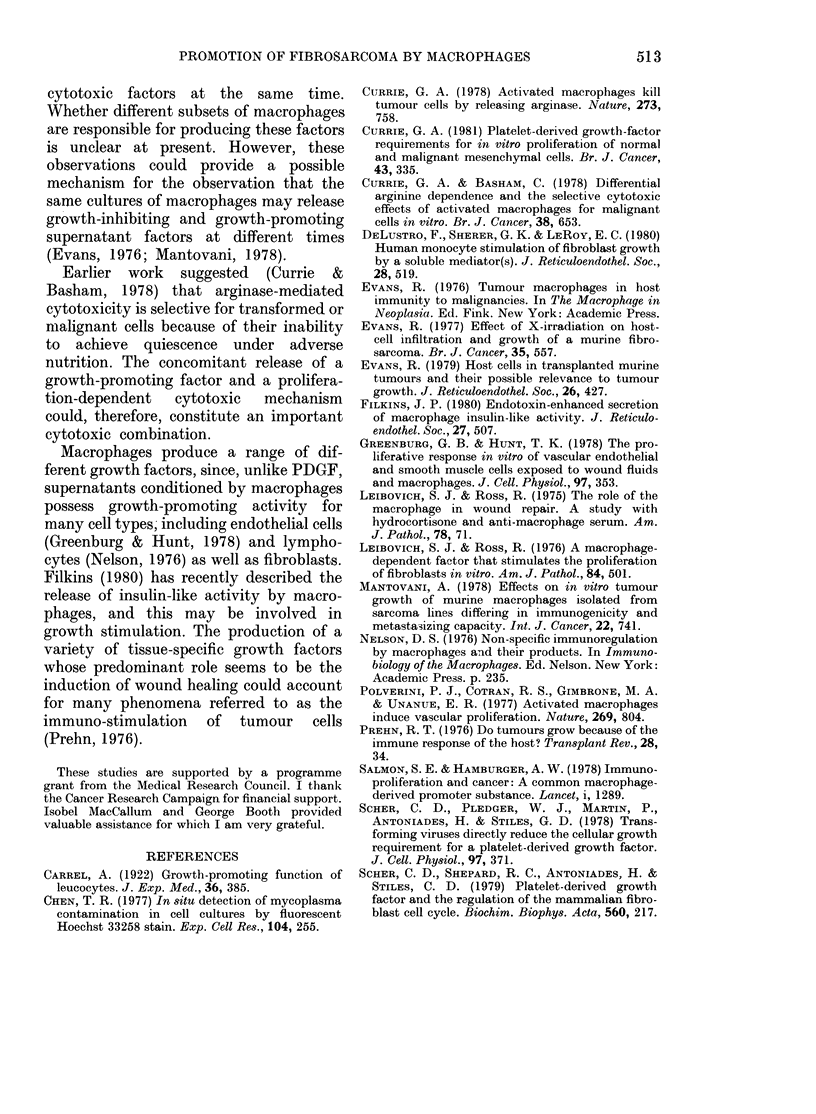# Promotion of fibrosarcoma cell growth by products of syngeneic host macrophages.

**DOI:** 10.1038/bjc.1981.219

**Published:** 1981-10

**Authors:** G. A. Currie

## Abstract

Cells from a C57BL/cbi chemically induced fibrosarcoma (FS6) require exogenous platelet-derived growth factor (PDGF) for in vitro proliferation (as do normal "untransformed" fibroblasts) whereas cells obtained from the FS6M1 tumour, a spontaneous metastasizing subline, show autonomy from PDGF in vitro. Furthermore, the FS6 cells exhibit very low colony formation in an anchorage-independent growth assay. In vivo, this tumour is immunogenic, rarely metastasizes and is heavily infiltrated by host macrophages. Studies of in vitro cell proliferation and anchorage-independent growth show that syngeneic host macrophages from the peritoneal cavity or from the growing tumour release a diffusible factor(s) which has (1) growth-stimulating activity on FS6 cells in monolayer cultures in PDGF-poor medium and (2) potent colony-stimulating activity on FS6 cell cultured in methyl-cellulose-containing medium. These macrophage supernatants stimulate proliferation of quiescent normal fibroblasts in monolayer culture as well as FS6 sarcoma cells, but do not stimulate anchorage-independent growth of normal cells. Supernatants from BCG-elicited macrophages were shown to contain abundant arginase, and were cytolytic to FS6 cells but not to normal cells. Heat inactivation abrogated the arginase and cytotoxicity, revealing heat-stable mitogenicity for FS6 cells and normal fibroblasts. The stimulatory effect of macrophages on FS6 sarcoma cells can be mimicked by the addition of the tumour promoter 12-tetradecanoyl-phorbol-13-acetate (TPA) and supports the hypothesis that macrophages could play a significant role in multistage carcinogenesis by providing a source of endogenous promoter.


*Br. J. Cancer* (1981) **44**, 506

### PROMOTION OF FIBROSARCOMA CELL GROWTH BY PRODUCTS OF SYNGENEIC HOST MACROPHAGES

G. A. CURRIE

*From the Division of Tumour Immunology, Chester Beatty Research Institute, Belmont, Sutton, Surrey*

Received 9 April 1981 Accepted 3 July 1981

**Summary.—Cells from a C57BL/cbi chemically induced fibrosarcoma (FS6) require exogenous platelet-derived growth factor (PDGF) for *in vitro* proliferation (as do normal "untransformed" fibroblasts) whereas cells obtained from the FS6M1 tumour, a spontaneous metastasizing subline, show autonomy from PDGF *in vitro*. Furthermore, the FS6 cells exhibit very low colony formation in an anchorage-independent growth assay. *In vivo*, this tumour is immunogenic, rarely metastasizes and is heavily infiltrated by host macrophages. Studies of *in vitro* cell proliferation and anchorage-independent growth show that syngeneic host macrophages from the peritoneal cavity or from the growing tumour release a diffusible factor(s) which has (1) growth-stimulating activity on FS6 cells in monolayer cultures in PDGF-poor medium and (2) potent colony-stimulating activity on FS6 cells cultured in methylcellulose-containing medium. These macrophage supernatants stimulate proliferation of quiescent normal fibroblasts in monolayer culture as well as FS6 sarcoma cells, but do not stimulate anchorage-independent growth of normal cells.**

**Supernatants from BCG-elicited macrophages were shown to contain abundant arginase, and were cytolytic to FS6 cells but not to normal cells. Heat inactivation abrogated the arginase and cytotoxicity, revealing heat-stable mitogenicity for FS6 cells and normal fibroblasts.**

**The stimulatory effect of macrophages on FS6 sarcoma cells can be mimicked by the addition of the tumour promoter 12-tetradecanoyl-phorbol-13-acetate (TPA) and supports the hypothesis that macrophages could play a significant role in multistage carcinogenesis by providing a source of endogenous promoter.**

IN 1922, Carrel showed that leucocytes from the blood or peritoneal cavity of chickens contain and release factor(s) which stimulate the growth of fibroblasts in tissue culture. Leibovich & Ross (1975, 1976) in studies of wound healing in guinea-pigs, suggested that macrophage infiltration may play a vital role in initiating the proliferation of fibroblasts, and they also showed that peritoneal-exudate macrophages maintained *in vitro* release a heat-stable non-dialysable fibroblast mitogen. Vascular proliferation in response to a macrophage product was described by Polverini *et al.* (1977) and macrophages derived directly from wounds were shown by Greenburg & Hunt (1978) to release potent mitogenicity for both endothelial and smooth-muscle cells. A potent mitogen for fibroblasts has been detected in cultures of human monocytes (DeLustro *et al.*, 1980).

The growth-promoting activity of macrophages has also been incriminated in the natural history of tumours. Evans (1977) studied a murine fibrosarcoma (FS6) and concluded that host macrophages infiltrating the growing tumour may in some unknown way promote its growth. He subsequently reviewed the topic (Evans, 1979) and argued that host macrophages may play a vital role in the development of tumours. Salmon & Hamburger (1978) suggested that macrophagederived factors may play such a role in promoting clonal proliferation of malignant cells, and could act as endogenous "promoters" analogous to the role of phorbol esters in multistage carcinogenesis.

While examining the proliferative capacity of cells from rodent mesenchymal tumours in plasma-derived serum (PDS) and their responses to platelet-derived growth factor (PDGF) we noted (Currie, 1981) that some tumours (including the FS6 sarcoma described by Evans, 1977) required an exogenous source of PDGF to proliferate *in vitro*. Furthermore, tumours found to be dependent on PDGF were those which, when implanted into the syngeneic host, attract large numbers of host macrophages, are immunogenic and rarely metastasize spontaneously (Currie, 1981).

The experiments described below lend weight to the hypothesis of Salmon & Hamburger (1978) and indicate that host macrophages infiltrating the FS6 sarcoma may stimulate tumour growth by releasing material mitogenic for FS6 sarcoma cells and for normal fibroblasts. Furthermore, such macrophage product(s) greatly enhance anchorage-independent growth of FS6 sarcoma cells but not of normal fibroblasts. The *in vivo* expression of the malignant phenotype by these cells may, therefore, be dependent upon "endogenous promotion" by products of infiltrating host macrophages.

### MATERIALS AND METHODS

*Fibrosarcomas.*—The FS6 fibrosarcoma, induced by benzo(a)pyrene in C57BL/cbi mice, was s.c. transplanted every 3 weeks using trocar fragments. All studies of this tumour were performed between *in vivo* Passages 5 and 9, and at Passage 9 were replenished from Passage 4 stocks held in liquid $N_2$. It is slow growing and rarely gives rise to distant metastases at these early passages. It is immunogenic in syngeneic mice by conventional transplantation-rejection criteria, and the growing tumour mass *in vivo* is heavily infiltrated by host macrophages. It was used and described by Evans (1977). The FS6M1 is a variant subline of the FS6 which arose during transplantation of FS6 in C57BL/6 mice in the laboratories of Dr A. Mantovani (1978). When transplanted back into its syngeneic host strain (C57BL/cbi) this tumour is weakly immunogenic, induces a high incidence of distant metastases and contains fewer host macrophages than the parent FS6. It was handled and transplanted in a manner similar to the FS6, and was used within 10 passages from its receipt back into these laboratories.

*Tumour macrophages.*—Fragments of FS6 tumour were washed, chopped and disaggregated with 0·1% collagenase (Type 1, Sigma) and 0·01% DNAse (Type 1, Sigma). The resulting cell suspension was filtered through gauze and washed in Hanks' balanced salt solution. The cells were counted, suspended at $10^6$/ml in arginine-free RPMI 1640 containing 10mM HEPES, penicillin, streptomycin and neomycin, and 5 ml was added to 60mm plastic Petri dishes. These cultures were incubated overnight at 37°C in 5% $CO_2$ in humid air. The cultures were then washed vigorously to remove dead and non-adherent cells. Examination of the resulting cell monolayers for Fc receptors by the addition of appropriately sensitized sheep erythrocytes revealed that over 95% of the cells possessed Fc receptors, and after reincubation at 37°C for 30 min were intensely phagocytic. These cells were trypsin-resistant, and when cultured in complete medium (see below) showed no evidence of proliferation.

*Peritoneal-exudate cells* were obtained by lavage from normal C57BL/cbi mice, or from mice which had previously received i.p. injections of 1 ml thioglycollate medium, 1 ml 10% proteose-peptone (4 days previously) or 300 $\mu$g Glaxo BCG (14 days previously).

*Plasma-derived serum* (*PDS*) *and whole-blood serum* (*WBS*) were obtained from normal healthy volunteers by methods previously described (Currie, 1981).

*Cell cultures.*—Enzyme-disaggregated cell suspensions from FS6 and FS6M1 (as above) were cultured in 25cm$^2$ plastic flasks in RPMI 1640 containing 10% heat-inactivated foetal bovine serum (FCS), antibiotics and HEPES (complete medium). They were passaged twice weekly using 0·1% trypsin, and were used between Passages 2 and 10, and were regularly replenished from Passage 2 stocks. The uncloned cultures from both tumours were tumorigenic when tested in syngeneic mice at Passages 2 and 10. Although it has not been assayed by radioimmunoassay, the batch of FCS contained high levels of PDGF by bioassay (unpublished).

The following cells were also used as targets in growth assays: $BHK_{21}C_{13}$ and PyY respectively, untransformed and polyoma virus-transformed lines of baby hamster kidney cells (from Flow Laboratories Ltd); $NLF_1$, a culture derived from an explant of chopped lung tissue of a male C57BL/cbi mouse is a characteristic normal fibroblast. These cells were also cultured in RPMI 1640 containing antibiotics, 20mM HEPES and 10% FCS. They were all grown as stock cultures in 25cm² disposable plastic flasks, and were passaged frequently to maintain exponential growth. They were routinely screened for mycoplasma contamination by Bisbenzimide H33258 fluorescence (Chen, 1977) and were negative throughout these experiments. All cell lines were studied within 10 *in vitro* passages and were regularly replenished from low-passage stocks.

*Cell-proliferation assay.*—The assay was performed exactly as described earlier (Currie, 1981). In brief, $2 \times 10^3$ cells were grown in 100μl volumes in Microtest II plates and cell numbers estimated at intervals on formalin-fixed cells, using a methylene-blue binding assay read in a Multiskan 8-channel photometer. Growth curves (as absorbance at 650 nm, A650) were plotted on log paper and cell-population doubling time measured. Sextuplicate wells were examined at each observation. The Multiskan was blanked on control wells which had contained cell-free medium. Data from this assay were also used to derive figures for percentage growth at 48 h; *i.e.* data from the test samples were compared to the nil control and any percentage increase (or decrease) in optical densities calculated as percentage change.

$$\% \text{ Growth} = \frac{\text{A650 test at 48 h} - \text{A650 at Time 0}}{\text{A650 at Time 0}} \times 100$$

*Arginase assay.*—Levels of arginase in the culture medium were assayed by a radioisotope method using $^{14}C$-guanidino-arginine. Details of this method will be published separately (Ould & Currie, in preparation).

*Anchorage-independent growth.*—Cells were suspended in complete medium containing 1·2% methylcellulose (Methocel A4M, Dow Chemical Co.) at $2{\cdot}5 \times 10^2$ or $2{\cdot}5 \times 10^3$/ml and 4 ml poured into 60mm plastic Petri dishes containing a 2ml underlay comprising 0·6% LGT agarose (Marine Colloids Inc.) in complete medium.

For some experiments $\sim 2 \times 10^6$ macrophages (proteose-peptone elicited or obtained from the FS6 tumour) were plated in the Petri dishes in complete medium and allowed to adhere at 37°C for 2 h. The dishes were then vigorously washed free of non-adherent cells and the remaining macrophage monolayer overlayed with 0·6% agarose which was allowed to gel at room temperature before target cells in 1·2% methylcellulose were added. Control-culture underlays of $10^6$ $BHK_{21}C_{13}$ cells, FS6 or FS6M1 cells were irradiated with X-rays under aerobic conditions to a dose of 35 Gy before the addition of target cells in methylcellulose. The cultures were incubated for 14 days and the colonies counted after staining *in situ* with iodonitrotetrazolium violet. The results are expressed as plating efficiency (*i.e.* the number of colonies counted as a percentage of the number of target cells added PE).

Before staining the dishes, sample single colonies were aspirated with a fine pipette under stereomicroscopy, transferred to culture flasks containing 5 ml complete medium and allowed to grow as monolayers. These clones were subsequently retested for anchorage-independent growth as described above.

### RESULTS

### *Growth of FS6, FS6M1, $NLF_1$ and $BHK_{21}C_{13}$ in PDS or WBS in monolayer cultures*

When cultured in RPMI 1640 containing 15% heat-inactivated WBS all 4 cell lines grew equally well. However, in 15% PDS the $BHK_{21}C_{13}$ cells, $NLF_1$ and FS6 failed to proliferate and remained quiescent; but the FS6M1 grew as well in PDS as in WBS. Growth curves of FS6 and FS6M1 are illustrated in Figs 1 & 2. When an extract of human platelets (containing 5 u PDGF, Currie, 1981) was added to FS6, $NLF_1$ or to $BHK_{21}C_{13}$ in PDS they grew rapidly: *i.e.* cells were dependent upon exogenous growth factor, as were the normal lung fibroblasts and normal ham-

FIG. 1.—Growth curves of FS6 and FS6M1 cells in medium containing plasma-derived serum (PDS). The results are shown as absorbance at 650 nm, and are the means of sextuplicate observations. Bars indicate total data spread.

FIG. 2.—Growth curves of FS6 cells in PDS to which was added control medium (●—●), platelet extract (○—○) and supernatant from FS6 tumour macrophages (○---○).

ster cells. Growth of FS6M1 was unaffected by the addition of platelet extract.

*Effect of macrophage supernatant on the growth of FS6 cells in monolayer cultures*

About $5 \times 10^6$ macrophages obtained as described above were cultured in 4 ml serum-free RPMI 1640 medium for 48 h and then the supernatants were collected,

TABLE I.—*Effect of macrophage supernatants on doubling time of FS6 cells in PDS in monolayer culture*

| Macrophage supernatant | Cell-doubling time (h) |
|---|---|
| Nil (control) | > 100 |
| Platelet extract (5 u) | 14·2 |
| Normal peritoneal | 18·4 |
| Proteose–peptone elicited | 14·9 |
| Proteose–peptone elicited and exposed to LPS (10 μg/ml) | − 17·2* |
| FS6 tumour macrophages | 15·0 |
| BCG elicited | − 21* |
| BCG elicited (heated) | + 13·6 |

* Negative value represents lysis of cells.

centrifuged and filtered through an 0·22-μM filter (Millipore). Fifty-μl volumes of these supernatants, plus control media from flasks incubated without cells, were added to wells containing 50μl volumes of RPMI 1640 medium containing FS6 cells and 30% PDS, *i.e.* a final concentration of 15% PDS. All these experiments were conducted in sextuplicate, and growth curves were constructed as before. The results are shown in Table I and Fig. 2. As the data show, the supernatant from normal resident peritoneal macrophages induced proliferation of FS6 cells. Supernatants from FS6 tumour-derived macrophages also contained powerful growth-promoting activity, providing cultures of FS6 cells which proliferated as rapidly as those exposed to platelet-derived growth factor. Peritoneal macrophages obtained from mice injected with thioglycollate provided supernatants with no detectable growth-promoting activity, whereas those elicited with proteose-peptone provided very actively growth-promoting supernatants. Macrophages elicited with BCG provided cytotoxic supernatants killing FS6 and FS6M1 cells. The normal cells were unaffected in that there was no evidence of either growth promotion or cytotoxicity. Proteose-peptone-elicited macrophages exposed to 10μg/ml lipopolysaccharide (Difco, *E. coli* 0555:B5) also provided cytotoxic supernatants.

*Effect of macrophage supernatant on $BHK_{21}C_{13}$ cells in monolayer culture*

As can be seen from Table II, supernatant media from normal peritoneal macrophages (those elicited by proteose-peptone or obtained within a growing FS6 tumour) provided potent mitogenicity for the $BHK_{21}C_{13}$ hamster fibroblasts. However, supernatants from macrophages elicited by BCG, or proteose-peptone macrophages exposed to LPS endotoxin, had no mitogenic effect on $BHK_{21}C_{13}$ cells. They had no detectable cytotoxicity on these cells, unlike their effects on FS6 cells.

TABLE II.—*Effect of macrophage supernatants on doubling time of $BHK_{21}C_{13}$ cells in PDS in monolayer culture*

| Macrophage supernatant | Cell-doubling time (h) |
|---|---|
| Nil (control) | > 100 |
| Platelet extracts (5 u) | 9·4 |
| Normal peritoneal | 14·5 |
| Proteose–peptone | 13·0 |
| Thioglycollate | > 100 |
| BCG elicited | > 100 |
| Proteose–peptone + LPS (10 μg/ml) | > 100 |
| FS6 tumour macrophages | 9·6 |

*Arginase activity in macrophage supernatants*

The various macrophage supernatants tested for growth-promoting activity were also assayed for arginase content. Low levels of arginase were present in all the media containing growth-promoting activity. However, cytotoxic supernatants (from BCG-elicited macrophages) contained high levels of arginase (see Table III).

*Heat stability of macrophage-derived growth factors*

Supernatant media from FS6 tumour macrophages were heated in a water bath to 100°C for 10 min. They were then centrifuged, filtered and tested for growth-promoting activity on FS6, FS6M1 and the "normal" cells, $NLF_1$ and $BHK_{21}C_{13}$. Despite this vigorous treatment the growth-promoting activity of these supernatants was not decreased; indeed there was evidence of an increase. Since arginase is heat labile, the cytotoxic supernatants from BCG-elicited or LPS-treated macrophages were similarly heated and tested. The cytotoxicity activity of these supernatants was abolished by heating, and growth-promotion became detectable. In other words, the growth-promoting activity of these supernatants had been masked by their cytotoxic arginase content. Heating led to a dramatic reduction in their arginase content (Table III).

*Anchorage-independent growth*

$BHK_{21}C_{13}$ cells produced less than 0·01% PE when cultured in methylcellulose-containing medium. FS6 sarcoma cells produced colonies, but their PE was very low (see Table IV). FS6M1 cells showed a high PE (see Table V). When cultured over a macrophage monolayer the PE of FS6 cells was greatly enhanced, whereas FS6M1 were unaffected. Peritoneal-exudate macrophages elicited by proteose-peptone, and macrophages obtained directly from the FS6 sarcoma, both exhibited this potent stimulation on FS6 cells. Macrophage underlays, from either source, showed no promoting effect on

TABLE III.—*Arginase content of macrophage supernatants and their effects on growth of FS6, FS6M1, $NLF_1$ and $BHK_{21}C_{13}$ cells. Negative values represent cell lysis*

| Macrophage supernatant | Arginase content (μM urea/min/ml) | Target cells (% growth) FS6 | $FS6M_1$ | $NLF_1$ | $BHK_{21}C_{13}$ |
|---|---|---|---|---|---|
| Proteose-peptone induced | 8·54 | +210 | 0 | +168 | +218 |
| BCG induced | 27·40 | −63 | −82 | 0 | 0 |
| BCG induced (heated) | 2·91 | +215 | 0 | +170 | +246 |
| FS6 tumour macrophages | 6·3 | −190 | 0 | +140 | +204 |
| FS6 tumour macrophages (heated) | N.D. | +245 | 0 | N.D. | N.D. |

TABLE IV.—*Effect of macrophage underlays on colony formation of FS6 cells in methylcellulose*

| Underlay | PE | Subculture PE |
|---|---|---|
| Nil | 0·03 | 0·02 |
| Proteose–peptone peritoneal macrophages | 28 | 0·02 |
| FS6 tumour-derived macrophages | 14·1 | 0·03 |
| FS6 sarcoma (35 Gy) | 0·02 | N.T.* |
| $BHK_{21}C_{13}$ (35 Gy) | 0·02 | N.T.* |

* N.T. = not tested.

$BHK_{21}C_{13}$ which continued to show a very low PE. The enhanced anchorage-independent growth of FS6 was reversible, since clones derived from colonies from these dishes reverted to a low PE when retested without a macrophage underlay (Table IV). Underlays of $BHK_{21}C_{13}$, FS6 or FS6M1 had no effect on colony formation by FS6 cells.

TABLE V.—*Effect af macrophage underlays on colony formation (PE) of FS6M1 and $BHK_{21}C_{13}$ cells in methylcellulose*

| Underlay | FS6M1 PE | $BHK_{21}C_{13}$ PE |
|---|---|---|
| Nil | 6·3 | < 0·01 |
| Proteose–peptone peritoneal macrophages | 6·6 | < 0·01 |
| FS6 tumour-derived macrophages | 6·4 | < 0·01 |

*Effect of TPA on anchorage-independent growth of FS6 cells*

The potent tumour promoter 12-tetradecanoyl-phorbol-13-acetate (TPA) was dissolved in acetone and diluted in medium. Equivalent concentrations of acetone were used in the controls. A range of concentrations of TPA was added to the medium of FS6, FS6M1 and $BHK_{21}C_{13}$ cells in methylcellulose and its effects on colony formation examined. As can be seen from Table VI, the added TPA had no effect on the normal fibroblasts ($BHK_{21}C_{13}$). Significant enhancement of colony formation by FS6 cells was seen at 10 and 1ng/ml TPA. Colony formation by FS6M1 cells was unaffected except at 100 ng/ml, which was inhibitory to both FS6 and FS6M1.

TABLE VI.—*Effect of macrophage supernatant and TPA on anchorage-independent growth of FS6, FS6M1 and $BHK_{21}C_{13}$ cells in methylcellulose*

| | | PE | | |
|---|---|---|---|---|
| Material added | | FS6 | FS6M1 | $BHK_{21}C_{13}$ |
| Nil | | 0·03 | 7·1 | < 0·01 |
| FS6 tumour-derived macrophage supernatant 1 : 5 | | 7·8 | 7·0 | < 0·01 |
| TPA (ng/ml) | 100 | 0·09 | 0·4 | < 0·01 |
| | 10 | 1·4 | 6·7 | < 0·01 |
| | 1 | 11·2 | 7·4 | < 0·01 |
| | 0·1 | < 0·01 | 1·0 | < 0·01 |

### DISCUSSION

The use of plasma-derived serum (PDS) for the investigation of tumours of mesenchymal origin has provided a useful approach to the examination of the role of "growth factors". In the absence of platelet-derived growth factor (PDGF) a potent heat-stable peptide hormone active at nM concentrations (Scher *et al.*, 1979) normal mesenchymal cells remain quiescent. Malignant transformation by SV40 virus is associated with reduced dependence on growth factors; *i.e.* with the acquisition of mitotic autonomy (Scher *et al.*, 1978). In a study of a series of rodent mesenchymal tumours it was found (Currie, 1981) that these sarcomas display a range of dependence on the "wound-healing hormone" PDGF. Cells obtained from tumours with minimal host-cell infiltrate, and which readily metastasize (such as FS6M1) proliferate without a source of exogenous PDGF, whereas others such as the FS6 remain quiescent without a source of growth factor. The correlation between growth-factor-dependent growth and biological behaviour of the tumours *in vivo*, including the extent of host-cell infiltration, suggested that host macrophages may play a role in promoting tumour-cell proliferation.

Evans (1977) examined the FS6 mouse sarcoma, and showed that whole-body irradiation of the host prevented host macrophage infiltration, and contrary to conventional expectation, this was associated with a delay in tumour growth. The experiments described here show that host macrophages infiltrating the FS6 tumour can release a potent heat-stable mitogenic material which, in monolayer cultures, can induce proliferation in quiescent normal hamster and mouse fibroblasts and FS6 tumour cells. These findings suggest that the host-cell infiltrate may play an important biological part in facilitating tumour-cell proliferation. Since macrophages seem to be important in inducing proliferation during wound healing, their activity in promoting the growth of some tumour cells can be regarded as an aberrant form of wound healing.

Mantovani (1978) has previously examined supernatant fluids from cultures of macrophages obtained from FS6 tumours, and demonstrated either inhibited or enhanced incorporation of labelled thymidine by FS6 cells, depending on the duration of the cultures. Unfortunately, the FS6 was not grown in syngeneic mice, so the significance of his observations must remain in doubt. Evans (1979) also examined FS6 tumour macrophages, and showed that spent medium obtained from such cells stimulated proliferation of murine lymphoma cells in sub-optimal culture conditions.

Although mouse macrophages obtained from the peritoneal cavity or from within the FS6 tumour, release a product which has potent growth-promoting activity for normal hamster cells ($BHK_{21}C_{13}$) in PDGF-deficient monolayer cultures, they had no effect on the capacity of these cells to form colonies in methylcellulose-containing medium. They did, however, exhibit potent colony-stimulating activity when tested on FS6 fibrosarcoma cells. Furthermore, an underlay of such macrophages (from the FS6 tumour or elicited from the peritoneal cavity of normal mice by proteose-peptone) released a diffusible factor which conferred a high colony-forming efficiency on FS6 cells, but not on the normal hamster cells. The FS6M1 subline cells already showed anchorage-independent growth which was unaffected by a macrophage underlay.

Anchorage-independent growth is often regarded as an important feature of the malignant phenotype. But the expression of this particular phenotypic feature by FS6 cells is conditional upon the presence of a product of host macrophages. Furthermore, its expression is reversible; colonies picked from macrophage-enhanced FS6 cultures provided clones with a low PE. Furthermore, the addition of the known tumour promoter TPA to cultures of FS6 cells in methylcellulose also induced a dramatic, but again reversible, increase in anchorage-independent growth by these cells. The high colony-producing subline FS6M1 was unaffected by TPA, except at high (toxic) concentrations. The similarities in biological effect of TPA and a factor released by host infiltrating macrophages raises the possibility that macrophages may play a key role in the development of tumours by providing a source of endogenous promoter. Chemically induced sarcomas, heavily infiltrated by host macrophages, may represent just one stage in tumour progression. *In vivo* passage of the FS6 sarcoma, and other similar rat and mouse sarcomas, usually leads to altered biological behaviour, *e.g.* enhanced spontaneous metastasis associated with reduced host macrophage infiltration.

Macrophages elicited by injection of mice with BCG, or exposed *in vitro* to lipopolysaccharide bacterial endotoxin, were in these experiments growth inhibitory to normal hamster fibroblasts and lytic to the mouse fibrosarcoma cells FS6 and FS6M1. These toxic supernatants contained high levels of arginase (Currie, 1978) and lost their toxicity and arginase activity after heat inactivation. After heating, these cytotoxic supernatants showed substantial growth-promoting activity, indicating that the macrophage populations studied were releasing heat-stable growth-promoting and heat-labile cytotoxic factors at the same time. Whether different subsets of macrophages are responsible for producing these factors is unclear at present. However, these observations could provide a possible mechanism for the observation that the same cultures of macrophages may release growth-inhibiting and growth-promoting supernatant factors at different times (Evans, 1976; Mantovani, 1978).

Earlier work suggested (Currie & Basham, 1978) that arginase-mediated cytotoxicity is selective for transformed or malignant cells because of their inability to achieve quiescence under adverse nutrition. The concomitant release of a growth-promoting factor and a proliferation-dependent cytotoxic mechanism could, therefore, constitute an important cytotoxic combination.

Macrophages produce a range of different growth factors, since, unlike PDGF, supernatants conditioned by macrophages possess growth-promoting activity for many cell types, including endothelial cells (Greenburg & Hunt, 1978) and lymphocytes (Nelson, 1976) as well as fibroblasts. Filkins (1980) has recently described the release of insulin-like activity by macrophages, and this may be involved in growth stimulation. The production of a variety of tissue-specific growth factors whose predominant role seems to be the induction of wound healing could account for many phenomena referred to as the immuno-stimulation of tumour cells (Prehn, 1976).

These studies are supported by a programme grant from the Medical Research Council. I thank the Cancer Research Campaign for financial support. Isobel MacCallum and George Booth provided valuable assistance for which I am very grateful.

### REFERENCES

CARREL, A. (1922) Growth-promoting function of leucocytes. *J. Exp. Med.*, **36**, 385.

CHEN, T. R. (1977) *In situ* detection of mycoplasma contamination in cell cultures by fluorescent Hoechst 33258 stain. *Exp. Cell Res.*, **104**, 255.

CURRIE, G. A. (1978) Activated macrophages kill tumour cells by releasing arginase. *Nature*, **273**, 758.

CURRIE, G. A. (1981) Platelet-derived growth-factor requirements for *in vitro* proliferation of normal and malignant mesenchymal cells. *Br. J. Cancer*, **43**, 335.

CURRIE, G. A. & BASHAM, C. (1978) Differential arginine dependence and the selective cytotoxic effects of activated macrophages for malignant cells *in vitro*. *Br. J. Cancer*, **38**, 653.

DELUSTRO, F., SHERER, G. K. & LEROY, E. C. (1980) Human monocyte stimulation of fibroblast growth by a soluble mediator(s). *J. Reticuloendothel. Soc.*, **28**, 519.

EVANS, R. (1976) Tumour macrophages in host immunity to malignancies. In *The Macrophage in Neoplasia*. Ed. Fink. New York: Academic Press.

EVANS, R. (1977) Effect of X-irradiation on host-cell infiltration and growth of a murine fibrosarcoma. *Br. J. Cancer*, **35**, 557.

EVANS, R. (1979) Host cells in transplanted murine tumours and their possible relevance to tumour growth. *J. Reticuloendothel. Soc.*, **26**, 427.

FILKINS, J. P. (1980) Endotoxin-enhanced secretion of macrophage insulin-like activity. *J. Reticuloendothel. Soc.*, **27**, 507.

GREENBURG, G. B. & HUNT, T. K. (1978) The proliferative response *in vitro* of vascular endothelial and smooth muscle cells exposed to wound fluids and macrophages. *J. Cell. Physiol.*, **97**, 353.

LEIBOVICH, S. J. & ROSS, R. (1975) The role of the macrophage in wound repair. A study with hydrocortisone and anti-macrophage serum. *Am. J. Pathol.*, **78**, 71.

LEIBOVICH, S. J. & ROSS, R. (1976) A macrophage-dependent factor that stimulates the proliferation of fibroblasts *in vitro*. *Am. J. Pathol.*, **84**, 501.

MANTOVANI, A. (1978) Effects on *in vitro* tumour growth of murine macrophages isolated from sarcoma lines differing in immunogenicity and metastasizing capacity. *Int. J. Cancer*, **22**, 741.

NELSON, D. S. (1976) Non-specific immunoregulation by macrophages and their products. In *Immunobiology of the Macrophages*. Ed. Nelson. New York: Academic Press. p. 235.

POLVERINI, P. J., COTRAN, R. S., GIMBRONE, M. A. & UNANUE, E. R. (1977) Activated macrophages induce vascular proliferation. *Nature*, **269**, 804.

PREHN, R. T. (1976) Do tumours grow because of the immune response of the host? *Transplant Rev.*, **28**, 34.

SALMON, S. E. & HAMBURGER, A. W. (1978) Immunoproliferation and cancer: A common macrophage-derived promoter substance. *Lancet*, i, 1289.

SCHER, C. D., PLEDGER, W. J., MARTIN, P., ANTONIADES, H. & STILES, G. D. (1978) Transforming viruses directly reduce the cellular growth requirement for a platelet-derived growth factor. *J. Cell. Physiol.*, **97**, 371.

SCHER, C. D., SHEPARD, R. C., ANTONIADES, H. & STILES, C. D. (1979) Platelet-derived growth factor and the regulation of the mammalian fibroblast cell cycle. *Biochim. Biophys. Acta*, **560**, 217.